# Supplementary material for: The feasibility of respondent-driven sampling with people who use drugs in rural Western Cape, South Africa: A qualitative study
Source: PLOS Glob Public Health. 2025 Jun 26;5(6):e0004065. doi: 10.1371/journal.pgph.0004065 (PMC12200662; doi:10.1371/journal.pgph.0004065)
Supplement: S1 Text — (PDF) [file pgph.0004065.s001.pdf]

Focus Group Disucssion Guide

Introduction:

1. We would like to know about whether people from [name of community] visit other neighbourhoods within Worcester?

Probe: which neighbourhoods do they visit, how often, and what are the reasons for these visits?

Probe: do any of these reasons include to buy or use drugs together and if so, can you tell us more about that?

Probe: how do people from [name of community] move around to other neighbourhoods within Worcester (walking, taxi, other vehicles)?

2. Tell me about people who hardly or never leave [name of community] -are there people for whom this is true?

Probe: What are some of the reasons for people who live in [name of community] rather staying in their neighbourhood? (language barriers, less stigma experience in own neighbourhoood)

Probe: what makes it difficult for people in [name of community] to travel to other neighbourhoods (e.g. safety, distance)? Are there any routes to other neighbourhoods, or specific neighbourhoods that may be difficult for people who live in [name of community] to reach?

2. In your opinion, what are people in [name of community] views on people who use drugs?

Probe: What do your neighbours, family and friends think about people who use drugs?

Probe: In your experience, how are people who smoke drugs treated in [name of community] ?

Probe: do you think gender influences how people who use drugs are seen or treated? Please tell me more about this?

3. What are some of the challenges for people who use drugs to tell other people about their substance use in this community?

Probe: Is this the same for everyone or are there some groups that may find it easier to talk about substance use?

Probe: Who would they usually confide in about their drug use? E.g. friends, family, other people who use drugs?

4. Tell us a little about the people in the community that you live in (name) who smoke drugs (tik, mandrax)?

Probe: who will people usually use tik with (age, gender etc)? And Mandrax?

Probe: Do people usually smoke these drugs with other people in [community name] people from other neighbourhoods in Worcester? How many people do they normally smoke with and how often? What may make it challenging to smoke drugs with people from neighbourhoods in Worcester other than [community name]?

Read: I want to tell you a little bit more about the activities we do with the people who are part of our study. If you have been part of our study already, you may already know some of this information. For the TOTAL study, people will come to the study site for a study appointment, lasting up to 2 hours. They will put on a mask when they enter the building and wear it for the duration of the appointment as a form of protection from infection, as will study staff. First, people will sign a consent form (like you did today). They will then provide a urine specimen to test for drug use. Following this, they will answer some questions administered by project staff, which includes questions about their drug use, how they feel, and finances within the home. They will then provide blood and sputum to test for HIV and TB disease. Each person will receive compensation of a 180 Rand grocery voucher for completing the appointment. They will then come back after about two week for a second appointment where they will receive the results of their TB test, which will take about 30 minutes. The way that people are recruited into their study is through a seed or person who is identified at the beginning of the study which we will talk about a bit later. From there, these seeds give other people who may smoke tik/Mandrax recruitment coupons to also take part in the study, and to continue recruitment.

General Logistics Questions

5. Our research site is based in the center of town in Worcester, very close to the McDonalds. Is this an area that people who use drugs from different Worcester neighbourhoods ever visit, and what are the reasons for this?

Probe: Do you think that this is an accessible location for a research site for the whole of Worcester? What about people from [community name] specifically?

Probe: Would you be able to get to the research site in the week for a study appointment on your own? What could some of the challenges be to visiting a site in town?

6. If you were taking part in a study, where would you suggest activities be held so that people who smoke drugs in [name of community] could easily attend study activities?

   Probe: Would our current site in Worcester be accessible to people who smoke drugs from [name of community]?

Probe: are gangs that may be active in particular areas/gang territories that may make it difficult for participants to travel in certain areas within Worcester?

Probe: Would people who smoke drugs from [name of community] want to visit a site based in [name of community] ? Why/Why not?

7. Can you describe some other reasons why certain people from [name of community] would not attend study appointments?

Probe: Can you think of any other reasons for why individuals who smoke drugs from [name of community] may not want to take part in this study?

8. For you and your friends or family from[name of community], when would be the best time to come to this study appointment at the clinic?

Probe: Would you be able to attend an appointment in the morning on any day of the week-which day would this be?

Probe: Can you tell us a bit more about conflicting responsibilities that may make it difficult to come to an appointment on a weekday morning?

Probe: How many hours do you think people who smoke drugs in [name of community] have to spend attending a study appointment? How long would an appointment be for it to be too long?

Study Recruitment

9. What do you think about using fingerprints to identify study participants?

   Probe: Will people from [name of community] be willing to do fingerprint readings to be able to enroll into the study?

   Probe: Can you think of any challenges that we may have with using fingerprint readings?

<u>Feedback on Peer Recruitment:</u> I now want to get your feedback on the peer recruitment strategy that we have been using to get people to participate in our study. At the beginning of the study, we will start with one person or seed. This person will be a person who is ≥15 years old, smokes tik and/or Mandrax, and lives in Worcester. They will come to the study site for their appointment. When they have finished their appointment, they will talk to two people they kl now who also

smoke these drugs and live in Worcester, give them a recruitment coupon and encourage them to make a study appointment. Those people will then come to the clinic for their appointment. Then they will each talk to two other new people who smoke drugs and live in Worcester and give them coupons. For each person successful recruited into the study, participants can receive a voucher. This means that people who smoke drugs find out about the study from other people who smoke drugs and live in Worcester.

10. We really want to include people in [name of community] in this study. Who would be the kind of person who would be able to help recruit their friends or others in their social circle that smoke

Probe:? What type of personality or characteristics should they have?

Probe: For people who are provided with recruitment coupons, what challenges do you think there may be to enroll into the study?

The person will also receive an incentive for every person they talk to who brings their coupon and completes their study appointment (up to 2 total). We will then give them an R60 grocery voucher for each person they recruited and who was successfully enrolled. They can either collect the vouchers during their second visit or come back later to collect their voucher(s).

11. Do you think people in [name of community] would be willing to talk to people that they know, and who smoke drugs, about the study for an extra grocery voucher worth 50 Rands?

Probe: Can you tell us a little bit about the type of person who you think you may hand your coupon to and is more likely to come to the study

Probe: In your opinion, where will these people be from? [name of community] or other neighbourhoods?

12. Let us talk about some of the challenges that may occur when trying to recruit potential participants for the study?

Probe: For example, who would you *not* give a recruitment coupon to and why not?

Probe: What are some of the reasons that it may take a long time for participants to hand out their coupons to other people who smoke drugs who may be eligible to participate in the study?

13. Do you think people who are in the study will come back for their second visit even if they have not given their coupons out, or people they have given coupons to have not enrolled in the study? How could we get people to come back to their second visit?

These are all of my questions that we will cover today. Thank you very much for taking the time to talk to us today. Does anyone have anything else they would like to say? Thank you for participating in the focus group today.
