# Supplementary material for: The feasibility of respondent-driven sampling with people who use drugs in rural Western Cape, South Africa: A qualitative study
Source: PLOS Glob Public Health. 2025 Jun 26;5(6):e0004065. doi: 10.1371/journal.pgph.0004065 (PMC12200662; doi:10.1371/journal.pgph.0004065)
Supplement: Attachment — Submitted filename: PLOS Global health review_bedingfield_Aug 26.docx [file pgph.0004065.s004.docx]

Friday, Aug 23, 2024

Dear Dr McDowell

Thank you for the opportunity to review this manuscript on the use of respondent driven sampling (RDS) to recruit people who smoke drugs in the western cape of South Africa to a research study on TB. In the manuscript the authors describe their work on a qualitative study where 10 focus groups were conducted to gather the descriptions of 84 participants on their drug use networks and perspectives on research participation in general and the use of RDS in particular. I congratulate the authors on this important work.

Overall, I recommend the manuscript be published in PLOS Global Health because the topic is important, the research is original, and methodological papers are highly valuable. Improving TB research to reach people who use drugs in South Africa is important for TB elimination. However, this manuscript requires a major re-write before reviewers can know if the manuscript meets other PLOS Global Health criteria for publication.

**Major feedback**

To know if the conclusions are supported by the data, specific objectives for the study need to be stated, so they can be aligned with the presented data, and authors’ conclusions. From my reading of the paper, I suggest some specific objective may be to: 1) describe the drug use patterns of PWSD in rural western cape, 2) describe the drug use networks of PWSD in rural western cape, 3) describe the perspectives of PWSD on participating in research and the appropriateness of RDS as a recruitment strategy in their community, 4) describe the perspectives of PWSD on barriers to using RDS and recommendations for overcoming barriers to RDS in their community, 5) generate data to improve sampling for ‘xyz’ research project.

To support the objectives, the authors should revise the introduction to explain what they see as the gap in the research filling this for TB research. In the introduction, the authors describe the value of RDS as a method of recruitment for TB screening ***and*** as a method of recruitment for research. Thus, is it not clear which is the focus on the paper and if and how they are connected. The results presented are focused on research, but even here, it is not clear whether drug networks and drug use patterns are included for the purpose of describing how TB is spread in these communities or for describing how drug use networks would affect research recruitment and sampling (i.e., strategy could facilitate a larger sample or strategy would recruit a diverse sample with representatives from various networks). Some of this information is in lines 404- 423 of the discussion. If the focus of the paper is indeed on the application of the method for research (i.e., how using RDS will improve TB research by recruited a larger and more diverse sample), the authors should clearly state this and what they see as the value of data generated with this technique to improving TB care.

Finally, the authors use the word formative throughout the manuscript. Formative research can take many different forms. For the purpose and the methods of the article to be understood by the readers, the authors need to 1) briefly describe the main research project, 2) describe when and how these data were collected in relation to data collected for the main research project, 3) describe how researchers used the data to improve the main research project.

**Section by Section // Minor feedback**

Abstract

1. As above may be better not to use the word formative at all, since there is not space to clearly explain what is meant.. instead explain objectives more clearly (including relationship to the main study)
2. As above, try to specify more specific objectives given space constraints.
3. Replace use of Worcester in the abstract with “rural area” as this is more significant for the average reader who not familiar with S. African geography.
4. Line 57 – as written this sounds like results. Author should state these were the topic of the FGD discussion guides.
5. Also need to include sampling/recruitment strategy for FGDs in methods.
6. Line 61 – see comment below about drug use networks vs social networks. Also the phrase “drug use social networks across… differences” does not make sense.
7. Line 65 - Conclusions should be written to be in the voice of the authors and not the participants. See comments above regarding clarifying the role of formative research in this study/study setting.

Introduction

8. Line 76 and 79—evidence requires context, how does this compare to other countries? Is this high?
9. Line 89- example of mix up between care and research- (see above)
10. Line 92- typo “gather” repeated
11. Line 98 and 100 typos.. ? delete “for participants”
12. Line 115 – see above regarding need to better explain the impact of RDS on the sample recruited/not recruited. What are some weaknesses of RDS and suggested ways to overcome the weaknesses (ie limiting to small social circles, unless there is a link to a wider network… )

Methods

13. Study design- Line 148- See above regarding connection to larger study. Could you reference a published protocol?
14. Include rationale for why FGD chosen, given the sensitive, stigmatized nature of the topic.
15. Was any specific methodology or theory used? Please further describe the research team for this study. What was eligibility criteria? Who screened for eligibility? How was it done?
16. What was sampling strategy and recruitment method for this study?
17. Table 1 should be in results
18. Line 160- specify if consent was verbal or written
19. Line 165 –more detail is needed.. transcribed in which language? Presumably “professional service” did translation from Afrikaans or isiXhosa to English? Was professional service AI or transcriptionist? Who did the back translation back to Afrikaans/IsiXhosa
20. More about topic guide development—What were the goals of the interview guide. Were the goals settled on because of a gap in the literature? Experiences with the larger research project? How was the community consulted? How was this feedback incorporated into the guide?
21. Analysis – More detail is required. Was coding done inductively? Were any predefined categories or theories used?? Only first level coding is described. What was the goal of the analysis? How were codes reduced and themes developed? How many were analyzed in duplicate and how many were done independently? How was kappa calculated if they remaining 8 were done independently? How was community consulted with results?

Results

In general, themes should be presented to support revised objectives. Also, quotes are used well to support the themes (some italics on quotes are missing).

22. Line 194- Theme 1 – Here or elsewhere, it would be helpful if the authors can explain the difference between “drug networks” and social network and use these terms consistently throughout the paper. It seems the term social network is often used throughout the paper when drug use network would be more appropriate.
23. Theme 2- please lines 241- 250 suggest selecting data to illustrate participants perspectives on explain how stigma impacts research recruitment or eliminate these lines if no such data exist
24. Themes 3 and 4 may make sense (pending revised objectives) to combine these themes and presenting “location” as the suggested solution to over come crime and violence which are a challenge to the use of RDS

Discussion

Well written but should be revised to be in alignment with revised objectives for this study. In particular the description of how this study was intended to inform the main study.

Line 447- as above, connections between this study and the main study are not explained, so this comment is quite confusing. How was the FGD done before the study was started (i.e., what was the role of this “formative” research)
