# Supplementary material for: The feasibility of respondent-driven sampling with people who use drugs in rural Western Cape, South Africa: A qualitative study
Source: PLOS Glob Public Health. 2025 Jun 26;5(6):e0004065. doi: 10.1371/journal.pgph.0004065 (PMC12200662; doi:10.1371/journal.pgph.0004065)
Supplement: Attachment — Submitted filename: response to reviewers 15 November 2024.docx [file pgph.0004065.s005.docx]

**Responses to Review Comments**

The authors would like to thank the reviewers for their thorough read of the submitted manuscript as well as their thoughtful recommendations. We are pleased that the reviewers both saw merit in the importance of our qualitative work. In addition, we believe that the suggestions made will assist greatly with improving the manuscript, ensuring that it meets all of the criteria for publication in PLOS Global Health. Detailed responses to issues raised have been provided in the table below:

**Reviewer 1:**

| **Major Revisions** | **Response to Reviewer** |
| --- | --- |
| To know if the conclusions are supported by the data, specific objectives for the study need to be stated, so they can be aligned with the presented data, and authors’ conclusions. From my reading of the paper, I suggest some specific objective may be to: 1) describe the drug use patterns of PWSD in rural western cape, 2) describe the drug use networks of PWSD in rural western cape, 3) describe the perspectives of PWSD on participating in research and the appropriateness of RDS as a recruitment strategy in their community, 4) describe the perspectives of PWSD on barriers to using RDS and recommendations for overcoming barriers to RDS in their community, 5) generate data to improve sampling for ‘xyz’ research project. | Specific objectives have been added to the study aims on page 7 of the study. while these are similar to the suggestions of the reviewer, the authors have tweaked these slightly. |
| To support the objectives, the authors should revise the introduction to explain what they see as the gap in the research filling this for TB research. In the introduction, the authors describe the value of RDS as a method of recruitment for TB screening ***and*** as a method of recruitment for research. Thus, is it not clear which is the focus on the paper and if and how they are connected. The results presented are focused on research, but even here, it is not clear whether drug networks and drug use patterns are included for the purpose of describing how TB is spread in these communities or for describing how drug use networks would affect research recruitment and sampling (i.e., strategy could facilitate a larger sample or strategy would recruit a diverse sample with representatives from various networks). Some of this information is in lines 404- 423 of the discussion. If the focus of the paper is indeed on the application of the method for research (i.e., how using RDS will improve TB research by recruited a larger and more diverse sample), the authors should clearly state this and what they see as the value of data generated with this technique to improving TB care. | Sections of the introduction have been rewritten to highlight the gap in research early in this manuscript, and try and tie the aim of the paper together on Page 5 more coherently, together with the added, more specific study objectives. |
| Finally, the authors use the word formative throughout the manuscript. Formative research can take many different forms. For the purpose and the methods of the article to be understood by the readers, the authors need to 1) briefly describe the main research project, 2) describe when and how these data were collected in relation to data collected for the main research project, 3) describe how researchers used the data to improve the main research project. | We agree that the word formative is a broad term used to describe a number of different kinds of research. While we have indicated that this is qualitative, descriptive work throughout the manuscript, we have decided to retain the word formative because 1) this research was conducted to inform the larger RDS study which has now been referred to more clearly in the manuscript, as well as when this data was collected and 2) Johnston, a very prominent researchers in the area of RDS, refers specifically to formative work around RDS. In addition, while a few sentences have been added around how the main RDS study took the recommendations into account, we also wanted to leave this broad enough for other researchers who are considering undertaking an RDS survey. |

| **Minor Revisions** | **Response to Reviewer** |
| --- | --- |
| *Abstract* |  |
| 1. As above may be better not to use the word formative at all, since there is not space to clearly explain what is meant. instead explain objectives more clearly (including relationship to the main study) | We thank the reviewer for this comment, and have ensured that it is clear that the formative work takes the form of qualitative research. The objectives have also been explained, as mentioned in the major comments above. |
| 2. As above, try to specify more specific objectives given space constraints. | This has been added to the introduction. |
| 3. Replace use of Worcester in the abstract with “rural area” as this is more significant for the average reader who not familiar with S. African geography. | Thank you for this excellent suggestion, it has been changed in the abstract. |
| 4. Line 57 – as written this sounds like results. Author should state these were the topic of the FGD discussion guides. | This is now line 61 and the word guide has been added. |
| 5. Also need to include sampling/recruitment strategy for FGDs in methods. | A sentence has been added in lines 59 to 60 to include this. |
| 6. Line 61 – see comment below about drug use networks vs social networks. Also the phrase “drug use social networks across… differences” does not make sense. | This has been reworded because it is actually about social networks of people who use drugs, and trust that this now clarifies the terminology used. We have also rephrased the specific sentence mentioned. |
| 7. Line 65 - Conclusions should be written to be in the voice of the authors and not the participants. See comments above regarding clarifying the role of formative research in this study/study setting. | Since this is qualitative research, we wanted to be clear that this is not generalizable but rather from the perspectives of the particular participants that were included in the study. However, we have reworded this slightly as well to read more like the authors’ conclusions from the results. |
| *Introduction* |  |
| 8. Line 76 and 79—evidence requires context, how does this compare to other countries? Is this high? | The authors appreciate this comment made by the reviewer, and have added some recent information on methamphetamine use in South Africa compared to the rest of the continent. However, comparisons for methaqualone are more complicated because this often is lumped with other sedatives in global reports, and we therefore decided to rather report information that is available in the specific province where this study is conducted, for context. |
| 9. Line 89- example of mix up between care and research- (see above) | This section has been rewritten to address earlier concerns that the reviewer had. We trust that this is now clearer that RDS research can also lead to improved engagement in care by reaching this vulnerable group of participants by identifying various health needs. |
| 10. Line 92- typo “gather” repeated | This has been corrected on page 5. |
| 11. Line 98 and 100 typos.. ? delete “for participants” | We thank the reviewer for picking up on this grammatical error, and it has been deleted. |
| 12. Line 115 – see above regarding need to better explain the impact of RDS on the sample recruited/not recruited. What are some weaknesses of RDS and suggested ways to overcome the weaknesses (ie limiting to small social circles, unless there is a link to a wider network… ) | Additional information has been added on page 6. |
| *Methods* |  |
| 13. Study design- Line 148- See above regarding connection to larger study. Could you reference a published protocol? | The published protocol is cited as reference 22 in this section. |
| 14. Include rationale for why FGD chosen, given the sensitive, stigmatized nature of the topic. | The authors understand the reviewers’ concerns around conducting focus group discussions with this population. While due to space constraints additional information has not been added to the paper, the authors have extensive experience in conducting FGDs with people who use drugs in this setting, and ensured all participants were comfortable speaking in a group setting on the topics involved, which was further checked on the day and also re-iterated in the informed consent form. |
| 15. Was any specific methodology or theory used? Please further describe the research team for this study. What was eligibility criteria? Who screened for eligibility? How was it done? | No specific theory was used for this specific study. However, additional information has been added on the recruitment process on page 8, and the eligibility criteria have been made clearer. |
| 16. What was sampling strategy and recruitment method for this study? | This has been added on page 8. |
| 17. Table 1 should be in results | The authors agree, and have moved this to the results section. |
| 18. Line 160- specify if consent was verbal or written | We obtained written informed consent, and this has now been specified. |
| 19. Line 165 –more detail is needed. transcribed in which language? Presumably “professional service” did translation from Afrikaans or isiXhosa to English? Was professional service AI or transcriptionist? Who did the back translation back to Afrikaans/IsiXhosa | We did not use any AI services for transcription and have added additional information for clarity on this process on page 9. |
| 20. More about topic guide development—What were the goals of the interview guide. Were the goals settled on because of a gap in the literature? Experiences with the larger research project? How was the community consulted? How was this feedback incorporated into the guide? | Details on this guide have been added on page 9, and the protocol paper is also referenced in this section for additional information. |
| 21. Analysis – More detail is required. Was coding done inductively? Were any predefined categories or theories used?? Only first level coding is described. What was the goal of the analysis? How were codes reduced and themes developed? How many were analyzed in duplicate and how many were done independently? How was kappa calculated if they remaining 8 were done independently? How was community consulted with results? | The authors agree that additional information is needed here and have now added this in the data analysis section. |
| *Results* |  |
| In general, themes should be presented to support revised objectives. Also, quotes are used well to support the themes (some italics on quotes are missing). | The themes have been reworked to take the reviewers’ comments into account. The manuscript has also been checked to ensure that themes are presented consistently. |
| 22. Line 194- Theme 1 – Here or elsewhere, it would be helpful if the authors can explain the difference between “drug networks” and social network and use these terms consistently throughout the paper. It seems the term social network is often used throughout the paper when drug use network would be more appropriate. | The authors are referring to the social networks of drug use and have amended terminology accordingly. Social network is a term that is, however, used widely in the RDS literature. |
| 23. Theme 2- please lines 241- 250 suggest selecting data to illustrate participants perspectives on explain how stigma impacts research recruitment or eliminate these lines if no such data exist | A few additional words have been added here on page 15, as the stigma is more about not wanting to disclose drug use. |
| 24. Themes 3 and 4 may make sense (pending revised objectives) to combine these themes and presenting “location” as the suggested solution to overcome crime and violence which are a challenge to the use of RDS | Location and safety has been combined into the barrier theme as a sub-theme due to the widespread discussion of this concern. |
| *Discussion* |  |
| Well written but should be revised to be in alignment with revised objectives for this study. In particular the description of how this study was intended to inform the main study. | This has been added throughout the discussion section, and the authors thank the reviewer for the opportunity to make the link between the formative, qualitative work and the main study clearer to readers of the manuscript. |
| Line 447- as above, connections between this study and the main study are not explained, so this comment is quite confusing. How was the FGD done before the study was started (i.e., what was the role of this “formative” research) | The authors have tried to clarify this connection in the discussion, but also earlier in the manuscript so that this is clear from the outset for readers. |

**Reviewer 2:**

| **General Comments** | **Response to Reviewer** |
| --- | --- |
| This is an interesting paper, and I congratulate the authors. Aside of its relevance with regard to testing a methodology, the paper gives great insight of what may be viewed as an emic view of this group as they live, create and mold relationships, and meet their drug needs while navigating the penal system and the public moral terrain, and their own identities as citizens and human beings. The paper shows a rich side to this groups lifeworld, desires, vulnerabilities, and resiliencies. I am not sure how this framing could be woven into this paper, so that these rich lives of a group pursuing agency are foregrounded, and how effort to bring RDS does appear to offset (although at the same time it may disrupt positively) these lives. I would wish to see some interpretive texts in the findings and also another couple in the discussion. The issues such as refusal of fingerprints so that one may come in disguise and get another voucher demonstrate compellingly this group’s need for resources and invite a careful consideration of where the line might lie between reimbursing / incentivizing on one hand, and exploiting vulnerabilities on the other. | We thank the reviewer for recognizing the value of this manuscript and are in agreement that this is a key population of people who use drug in a rural setting that have a number of competing needs, life challenges and complex relationships. In summary, we agree that it is critical to depict our participants in a non-stigmatising way, without judgement. In fact, we are submitting a separate paper to highlight the types and levels of stigma that people who use substances and the effects that this has on their mental and physical health, because this warrants a rich description. However, the point of taking into account resources and incentives has particularly been noted. Therefore, a paragraph has been added on the complexity of this group on page 22 of the discussion section. |
| **Minor Revisions** | **Response to Reviewer** |
| *Abstract* |  |
| 1. Use respondent-driven or respondent driven consistently | We thank the reviewer for this catch and have conducted a global search to ensure respondent-driven sampling is used throughout the manuscript. |
| 2. Ten FGD (n=84) – how ‘n’ is used here is not clear; may I suggest specifying in this way or similar ‘total number of participants’ = 84. | This has been added to the methods section of the abstract. |
| 3. You could specify the kind of place or level of organization Worcester is in SA (city, province, suburb? | To be consistent with the comments also made by Reviewer 1, we have added that this is a rural town. |
| 4. Specify in the abstract how you actually recruited these participants, and their demographics too. | A sentence on recruitment procedures has been added to the methods section of the abstract. In terms of space constraints, we were unable to add the demographics but there is a table included in the submission where this is available in detail. |
| 5. In the results: I would suggest specifying the number and areas of themes first (before starting to describe actual results as has been done in the opening line). | We appreciate this comment, and have made some slight adaptations to the results section, but again word limit constraints prevent too much detail from being included in the abstract. |
| 6. I would like to suggest not just listing the themes, but actually summarizing key findings therein, to help your conclusions and findings to align. | As with the previous comment, this is a valuable comment, and have made some slight adaptations to the results section, but again word limit constraints prevent too much detail from being included in the abstract |
| 7. Specify, as you do in the main paper, that the recommendations to enhance the uptake of RDS by PWSD’, came from participants, not from the researchers. | This has been added to the conclusion section of the abstract. |
| *Manuscript Text* |  |
| 1. Line 75: Please give citation for household survey | The citation for this survey has been provided in the text and added to the references. |
| 2. Check grammar – “as a potentially feasible methods” | The grammatical error has been corrected. |
| 3. Grammar Ln 99: “and it known for its efficiency and cost effectiveness“ | The word “is” has been added to correct the grammar of this sentence. |
| 4. Ln 100: Not clear what “and potentially for participants” is referring to | The authors agree with this comment and have deleted this phrase from the manuscript. |
| 5. Ln 101 “PWSD urban communities of PWSD” | This has been revised to PWSD in urban communities. |
| 6. Ln 105. You refer to ‘increased stigma’ when talking about rural areas; did you have citations that point to this? | A few sentences have been added (see page 5) on the issue of increased stigma in rural areas in the text, as well as references as evidence for this claim. |
| 7. Ln 109: please check “97,00” | This was indeed an incorrect number and has been changed to 97,000. |
| 8. Since you describe the demographic composition of the study, could you provide some breakdown of subgroups living there | Some additional information on the demographic breakdown of people who live in Worcester has been added to page 6. |
| 9. Line 122.You mention one of the requirements of RDs as ‘ensuring that the population of interest (for example PWSD) is adequately socially networked and allows relationships of different strengths and types to ensure social diffusion” Could you please elaborate regarding social diffusion, and the context in which you refer to the specific paper (the paper does not seem to explicitly mention social diffusion). | This is a term mentioned by Johnston in a well-cited article on RDS. Additional information has been added to explain the concept of social diffusion. |
| 10. Ethical issues do not seem a little out of place under design. | The authors are in agreement with this comment and have moved this to a separate section at the end of Methods (page 10). |
| 11. Ln 147 – when you say historically disadvantaged, could you please specify for an international audience. | This has been modified on page 8 to read economically disadvantaged, and information on the legacy of apartheid added. |
| 12. In the paragraph on recruitment (Ln 145-154), if you could specify who was screened for eligibility, was it all potential participants (including those identified through friends from the study, and those identified by study staff by entering the community). | This has been rewritten to clarify that all potential participants were screened to ensure that they met eligibility criteria. |
| 13. The table is not included in the draft paper | Table 1 is added as a separate file to the manuscript. |
| 14. Ln 163 – Please elaborate on the significance of ‘Facilitators were not involved in participant recruitment and had no prior relationships with participants” | This is added on page 9 to avoid any kind of bias in data collection methods. |
| 15. Ln 167” Please elaborate how community input into the FGD guide was obtained | More detail has been added that this was input from a community advisory board and has been added on page 9. |
| 16. Ln 184 = “Two of the authors presented key findings to members of the community to ensure accuracy shortly after the majority of FGDs were held in 2020.” Was this after much of the analysis had been done, or after only partial analysis? | On page 10, the fact that these were preliminary results before the analysis process had been completed has been added. |
| 17. Could you please review the ordering of your first 2-3 paras of findings. You mention in para 1 causal relationships and larger networks of up to 160 people. However, a little further below (ln 208-212) you seem to imply you have not referred prior to casual groups. Could you try to find a way to tie this part together. | We appreciate the comment about the difference between more established and casual relationships in drug use networks. However, we have finessed the wording to make clear that both of these kinds of relationships were reported by participants in this study. |
| 18. The earlier part of the findings appears to be a form of mapping, and it might help to actually present these forms or relationships in a figure of some sort. I would understand, though, if it turned out to be difficult conceiving the figure. Please take this suggestion at your discretion. | The authors think that a figure is an excellent idea but have another manuscript that will focus on mapping out these social networks in more detail, and we have therefore decided not to include this kind of figure for this specific paper. |
| 19. Check spelling in ln 227 | This quote on page 12 has been corrected. |
| 20. Lines 231-234 are hard to follow, maybe due to the use of negatives. Please consider rephrasing. | The authors agree with the reviewers’ comments here and have rewritten this sentence so that it reads easier. |
| 21. Ln 264 Plea review the grammar, police car stand’. If ‘stand’ is verbatim, I would suggest using a more correct or common term in parenthesis. Also, what does ‘going to a study’ and ‘shake me out’ mean? | Additional information has been added in parenthesis to this quote so that readers can understand the participants’ vernacular better. |
| 22. Is there any plausible explanation for the PWUD not wanting to the evidence of their use in the bloods. | While there was some information on potential reasons for this, an additional line has been added on page 14. |
| 23. Please review quote in ln 326 -329 to make clearer | Additional information has been added in parenthesis to this quote so that readers can understand the participants’ vernacular better on page 16. |
| 24. Check grammar in paragraph starring 331 | This sentence has been rewritten. |
| 25. Among limitations you mention that discussions were conducted in the local language resulting in potential to lose themes during translation. As a limitation, this is not clear to me; often local language is encouraged, and the researchers would need to ensure staff are competent or translation is available. | The authors agree that it is incredibly important to allow participants to participate in study activities in the language which they feel most comfortable with, and indeed this was the case. However, direct translations into English are sometimes challenging from local languages, where some of the vernacular is quite specific. This has been explained in the limitations section in a clearer way. |
| 26. I also learnt towards the end of the paper, that the RDS seemed to already have been implemented at this time of submitting this paper. Is this correct? Reference to this seems to be in passing. It may help to make clearer much earlier that the RDS study was subsequently carried out, and that the full reporting of findings will be done separately. This may help to contextualized some of very rich and telling findings presented in this analysis. | The authors feel that this is an excellent point and have now spoken about the use of the formative, qualitative study findings to inform the larger RDS study methods throughout the manuscript. |
