# Supplementary material for: The feasibility of respondent-driven sampling with people who use drugs in rural Western Cape, South Africa: A qualitative study
Source: PLOS Glob Public Health. 2025 Jun 26;5(6):e0004065. doi: 10.1371/journal.pgph.0004065 (PMC12200662; doi:10.1371/journal.pgph.0004065)
Supplement: Attachment — Submitted filename: feasibility of RDS_ re-review Feb 20_nb.docx [file pgph.0004065.s006.docx]

Dear Dr McDowell

Thank you for this opportunity to re-review the manuscript titled "The feasibility of respondent-driven sampling with people who use drugs in rural Cape Town, South Africa: a qualitative study”. In this work, the authors describe a preliminary study conducted to gather the perspectives of people who smoke drugs in the Western Cape of South Africa on participating in research which incorporates respondent driven sampling. After revisions, the manuscript is much improved. Congratulations to the authors for their hard work on this important topic. However, I feel additional revisions are still required before the manuscript is suitable for publication. I outline my major and minor points of feedback below.

Major revisions

1. I found there was disconnection between the research objective, the titles of the themes, and the data presented. Much of the data presented does not seem to be about respondent driven sampling (RDS) at all, but about participation in research generally (e.g., location of the research, level of comfort with specific types of data collection, stigma of being a drug user). Thus, I suggest revising the objective statement and the titles of the themes to fit the data presented.

For the objective, I suggest specifying sub-objectives (along these lines):

a. To learn about the social networks of ppl who use drugs in this region
b. To learn about the feasibility, acceptability and barriers of using RDS in this this population
c. To learn about the feasibility, acceptability and barriers of research participation in this population

For the themes, I suggest revising to something like:

a) social network characteristics of PWSD
b) perspectives of PWSD on research participation and RDS methods
c) potential challenges to research participation and RDS use for PWSD
d) participant recommendations to enhance the uptake of research and RDS by PWSD
2. The manuscript needs a general edit for clarity. Many sentences are confusing and hard to follow. Several sentences seem to have extra words or are not focused on a clear point.
   a. Examples are at line 121 – 124 and lines 135- 137
3. The abstract will need to be updated to reflect changes to objectives, themes, etc

Specific and/or Minor revisions

1. Line 62- consider rephrasing “extensive drug use”- this sound as though participants are using a lot of drugs, which I don’t believe is the focus of the study. I believe that the focus should be that PWSD use drugs with different groups of people- which is good for RDS sampling
2. I suggest to bolster description of RDS at Line 103 – 105. I believe that RDS is a quantitative sampling method, which would be good to state explicitly, but is it more than a sampling method? It would also be helpful to more clearly say that with RDS, it is important that seeds are part of different social networks. Also, its important to describe the coupon system. What does the coupon entitle the recruiter to receive (money?)? What are they passing on the next recruit? Do the seeds receive more than one coupon?
3. Also having all the RDS information in one place would be less confusing, thus I suggest moving the sentences at line 121 to 126 and line 439-441 to around Line 103-105
4. At line 118 - It would be helpful to add one or two sentences about the main study and how results from this study informed that study. Also, the reference to the main protocol needs to be corrected throughout. I believe it should be # 22 not # 21. Also adding a sentence to specify “more information on the main study has been published elsewhere (i.e, 22) would be helpful
5. I suggest moving content at line 155 -157 to 131- 135 so all the background is in one place
6. Line 148 – how many FGDs for each language were held? Why were 2 more FGD’s added?
7. Can you include a bit more description and/or include examples of recruitment practices at lines 158- 161. Did the research assistants mention this study at study visits? How did the research assistants “do outreach”, did they go to places that drug users are known to congregate? After the potential participants were screened, did the research assistants tell people to go to a particular place at a particular time for the FGD? Told them to bring their friends?

Including this information will help the reader to understand rationale for number and size of the FGDs.

8. Line 165- Please add how many people were in each focus group.
9. Line 179- 180. To match with the data presented, you could also add that questions were directed to learning the feasibility of particular data collection and procedures planned for the larger study (i.e., blood and body fluid collection, fingerprints for ID checks)
10. Data analysis section – Please include here the feedback from the community when the initial coding was presented. Please also add a sentence to describe how the initial codes were developed into the ultimate themes. What kind of independent work and/or group discussion occurred?
11. Results at Line 258 – 264- the connections between this data, RDS sampling, and the main research project is not immediately clear. This could be addressed by adding information in the background section that the main study seeks to recruit PWID.
    a. Also the authors could rephrase the introduction to the quote by saying that one of the reasons mentioned by participants that PWSD do not use drugs with PWID is that they don’t go through the supply at the same rate or get the same effects.
12. As mentioned in major revision #1 - most of data in themes 2,3, and 4 do not seem to be about the recruitment method (RDS) specifically but participation in research in general. With respect to stigma, in my mind, it seems it would be less of an issue since the recruitment is happening within social networks where everyone uses drugs. Is there some nuance here between RDS recruitment specifically and general research participation which could be described? Either renaming this theme or explain in more detail the connection to RDS would be helpful.
13. Line 454-455 - Some of this information should be included in the introduction to help the reader understand the results.
14. Line 488- 489- Can you explain this limitation in more detail? For example, is it possible that participants were less engaged in the last 2 groups because they knew the methods had already been decided? Or possibly they had better answers to the FGD questions because they had already participated in the main study and could comment more specifically on what was actually done instead of imagining what might be done?
15. Line 494- You can add that high quality translation practices would mitigate this risk
