# Supplementary material for: The feasibility of respondent-driven sampling with people who use drugs in rural Western Cape, South Africa: A qualitative study
Source: PLOS Glob Public Health. 2025 Jun 26;5(6):e0004065. doi: 10.1371/journal.pgph.0004065 (PMC12200662; doi:10.1371/journal.pgph.0004065)
Supplement: Attachment — Submitted filename: response to reviewers April 2025.docx [file pgph.0004065.s007.docx]

**Responses to Reviewer Comments**

The authors would like to thank the reviewer for another detailed read of the submitted manuscript as well as careful considerations of the revisions made. We believe that the additional changes made will ensure that the manuscript meets all of the criteria for publication in PLOS Global Health. Detailed responses to issues raised have been provided in the table below:

**Reviewer 1:**

| **Major Revisions** | | **Response to Reviewer** |
| --- | --- | --- |
| 1. I found there was disconnection between the research objective, the titles of the themes, and the data presented. Much of the data presented does not seem to be about respondent driven sampling (RDS) at all, but about participation in research generally (e.g., location of the research, level of comfort with specific types of data collection, stigma of being a drug user). Thus, I suggest revising the objective statement and the titles of the themes to fit the data presented.  For the objective, I suggest specifying sub-objectives (along these lines):   a. To learn about the social networks of ppl who use drugs in this region b. To learn about the feasibility, acceptability and barriers of using RDS in this this population c. To learn about the feasibility, acceptability and barriers of research participation in this population   For the themes, I suggest revising to something like:   a) social network characteristics of PWSD b) perspectives of PWSD on research participation and RDS methods c) potential challenges to research participation and RDS use for PWSD d) participant recommendations to enhance the uptake of research and RDS by PWSD | We would like to take this opportunity to thank the reviewer for this comment. The comment made about the data not representing RDS has been addressed below.  The reviewer is correct in that RDS methodology is about sampling (recruitment of peers), but the sampling is part of bigger studies which most often collect biobehavioural data from key populations. Therefore, it is not about research participation in general, but rather research participation as part of the broader study which uses RDS recruitment methods.  The objectives and results have been slightly rewritten to include the larger study activities. However, we have not chosen to completely specify or change the objectives but rather add to the existing ones to ensure that this is clarified. In addition, part of RDS is successful peer recruitment, which means peers who smoke drugs are actually enrolled into the larger study, and this may be affected by additional factors outside of the peer group.  We have reworked the themes slightly to discuss study recruitment through RDS as well as participation in overall RDS study activities. We have also discussed the difference between seed participants, and peer recruitment of these seeds, in the results section more clearly. | |
| 2. The manuscript needs a general edit for clarity. Many sentences are confusing and hard to follow. Several sentences seem to have extra words or are not focused on a clear point.  Examples are at line 121 – 124 and lines 135- 137 | The authors appreciate this comment and have made a number of changes to the manuscript after reading it in detail. These mainly consist of grammatical changes to ensure that the manuscript is easy to follow. | |
| 3. The abstract will need to be updated to reflect changes to objectives, themes, etc | We have partly responded to this in the first point raised by the reviewer above. The abstract has been reviewed and revised to make it easier to read. | |

| **Minor Revisions** | **Response to Reviewer** |
| --- | --- |
| 1. Line 62- consider rephrasing “extensive drug use”- this sound as though participants are using a lot of drugs, which I don’t believe is the focus of the study. I believe that the focus should be that PWSD use drugs with different groups of people- which is good for RDS sampling. | We are in full agreement with this comment, and the word “extensive” has been removed and test has been added to show that these large social networks are favorable for RDS methods. |
| 2. I suggest to bolster description of RDS at Line 103 – 105. I believe that RDS is a quantitative sampling method, which would be good to state explicitly, but is it more than a sampling method? It would also be helpful to more clearly say that with RDS, it is important that seeds are part of different social networks. Also, its important to describe the coupon system. What does the coupon entitle the recruiter to receive (money?)? What are they passing on the next recruit? Do the seeds receive more than one coupon? Added information from line 103-106 here as requested | We thank the reviewer for this comment, and have added more information on the use of RDS as a recruitment method for larger study activities. In addition, we have added some more information on the peer recruitment process in Line 109-112. Since studies provide incentives in different ways (money, groceries, airtime etc) we have kept the phrase incentive as it encompasses all of these. |
| 3. Also having all the RDS information in one place would be less confusing, thus I suggest moving the sentences at line 121 to 126 and line 439-441 to around Line 103-105 | While we appreciate this suggestion, we were unsure what is meant here as line 439 is part of the result section. We have also structured the introduction to tell the story of not only as RDS as a recruitment method, but also one that is part of larger studies that often collect biobehavioral information. We have added information on RDS and larger study activities to ensure a better flow. |
| 4. At line 118 - It would be helpful to add one or two sentences about the main study and how results from this study informed that study. Also, the reference to the main protocol needs to be corrected throughout. I believe it should be # 22 not # 21. Also adding a sentence to specify “more information on the main study has been published elsewhere (i.e, 22) would be helpful | Thank you for this suggestion, we have added more details about the main study here. We have also corrected the reference throughout the manuscript. |
| I suggest moving content at line 155 -157 to 131- 135 so all the background is in one place | The additional contextual information for Worcester has been moved from the methods section to line 145-146 in the background. |
| 5. Line 148 – how many FGDs for each language were held? Why were 2 more FGD’s added? | Additional information has been added to line 157-159 to clarify the reviewers’ questions. |
| 6. Can you include a bit more description and/or include examples of recruitment practices at lines 158- 161. Did the research assistants mention this study at study visits? How did the research assistants “do outreach”, did they go to places that drug users are known to congregate? After the potential participants were screened, did the research assistants tell people to go to a particular place at a particular time for the FGD? Told them to bring their friends?  Including this information will help the reader to understand rationale for number and size of the FGDs. | A more detailed description his has been added between line 166-170.  In terms of number and size of the FGDs, we used traditional guidelines based on previous research and also extensive experience to have a minimum size of three, and a maximum size of ten, in focus group but these were also determined by language spoken and self-reported gender identity. |
| 7. Line 165- Please add how many people were in each focus group. | We would like to thank the reviewer for this request and have now added the range of participants in FGDs to line 171 to provide the reader with an indication of the size of these FGDs. However, after careful consideration we feel that it is too detailed to add each group size based on previous qualitative research published in this journal. We have also, as indicated above, followed general recommendations for FGD size |
| 8. Line 179- 180. To match with the data presented, you could also add that questions were directed to learning the feasibility of particular data collection and procedures planned for the larger study (i.e., blood and body fluid collection, fingerprints for ID checks) | This information has now been added to row 184 as requested. |
| 9. Data analysis section – Please include here the feedback from the community when the initial coding was presented. Please also add a sentence to describe how the initial codes were developed into the ultimate themes. What kind of independent work and/or group discussion occurred? | Explain that no new findings emerged, agreed with what was found in the FGDs in lines 215-217. |
| 10. Results at Line 258 – 264- the connections between this data, RDS sampling, and the main research project is not immediately clear. This could be addressed by adding information in the background section that the main study seeks to recruit PWID.  Also the authors could rephrase the introduction to the quote by saying that one of the reasons mentioned by participants that PWSD do not use drugs with PWID is that they don’t go through the supply at the same rate or get the same effects. | This has been revised in line 279-282, and there is also mention about PWID in the introduction. |
| 11. As mentioned in major revision #1 - most of data in themes 2,3, and 4 do not seem to be about the recruitment method (RDS) specifically but participation in research in general. With respect to stigma, in my mind, it seems it would be less of an issue since the recruitment is happening within social networks where everyone uses drugs. Is there some nuance here between RDS recruitment specifically and general research participation which could be described? Either renaming this theme or explain in more detail the connection to RDS would be helpful. | This has partly been addressed above. In terms of stigma specifically, this was not necessary about recruiting peers who also used substances but actually enrolling into the larger study as individuals were concerned about people outside of their social networks knowing about their drug use. The results have been slightly tweaked to make this clear. |
| 12. Line 454-455 - Some of this information should be included in the introduction to help the reader understand the results. | This has been added to the introduction as suggested. |
| 13. Line 488- 489- Can you explain this limitation in more detail? For example, is it possible that participants were less engaged in the last 2 groups because they knew the methods had already been decided? Or possibly they had better answers to the FGD questions because they had already participated in the main study and could comment more specifically on what was actually done instead of imagining what might be done? | We have included more information on this limitation in row 554-555. |
| 14. Line 494- You can add that high quality translation practices would mitigate this risk. | This is an excellent suggestion made by the reviewer, and we have now added this text to row 559 to 560. |
