# Supplementary material for: The feasibility of respondent-driven sampling with people who use drugs in rural Western Cape, South Africa: A qualitative study
Source: PLOS Glob Public Health. 2025 Jun 26;5(6):e0004065. doi: 10.1371/journal.pgph.0004065 (PMC12200662; doi:10.1371/journal.pgph.0004065)
Supplement: Attachment — Submitted filename: PLOS RDS_re-review May 1.docx [file pgph.0004065.s008.docx]

May 1, 2025

Dear Dr McDowell

Thank you for this opportunity to re-review the manuscript titled "The feasibility of respondent-driven sampling (RDS) with people who smoke drugs (PWSD) in rural Cape Town, South Africa: a qualitative study”. In this work, the authors describe a preliminary study conducted to gather the perspectives of PWSD in the Western Cape of South Africa on participating in research which incorporates respondent driven sampling. After revisions, the manuscript is much improved. Congratulations to the authors for their hard work on this important topic. I feel the manuscript is now suitable for publication, but several minor revisions, which are outlined below, are still required.

1. **Abstract**
   a. Line 52. Phrasing of the sentence is awkward. I suggest cutting “to conduct formative research” as it is not necessary to the meaning of the sentence and the word formative is mentioned in the conclusion
2. **Introduction**
   a. Line 103-108. The use of incentives remains unclear. I suggest the authors rephrase these lines to more clearly explain. Do all who recruit additional participants receive incentives? At what point of the process? If this varies for different studies, that could be described.
   b. Line 114- 129 – It remains is difficult to follow this paragraph as the requirements for RDS and the connection between this study and the main study are mixed together. For clarity, I suggest to move some of the sentences from lower in the paragraph to the start.
   c. Line 130 – the population for the study is not clearly described in the research question. I suggest to rephrase research question to “we aimed to explore the perceptions of PWSD in a rural region of the Western Cape on the feasibility and appropriateness of participation in a research studying using RDS.
   d. Line 139 – cut the word possibly- typo?
3. **Methods**
   a. Line 164- Sentence starting “participants were …” is long and confusing. I suggest breaking up by stating “Participant were recruited to the study in three ways. First…
   b. Line 183- Transcription and translation practices are not clearly described. I suggest “FGDs were transcribed verbatim in Afrikaans and isiXhosa by staff who spoke those languages. Afrikaans and isiXhosa transcripts were then translated into English
      i. Unless there was back translation to Afrikaans and isiXhosa, I suggest cutting the word back from this sentence.
      ii. What training was provided to the original transcribers ?
   c. Line 191 – I suggest rephrasing to “perspectives on study methods (e.g., peer recruitment, biomedical fingerprints)”. RDS practices are discussed lower down
   d. Line 195- Please describe the amount of incentive
   e. Analysis – Line 199 – 211 – methods for condensing codes into higher order themes are not described. Please add practices used to complete analysis.
4. **Results**
   a. Line 227- the word “characteristics” is repeated. Please cut one for clarity
   b. Line 325 – 337 – Although the stigmatized nature of drug use is an important consideration for this study, the authors do not describe the impact of this stigma on research participation. Please add one or two sentences to explain this link.
5. **Discussion**
   a. Sentence at line 482- as per point above. This is not clearly supported by the data. Further description of the results on this topic would address this.
   b. Line 520-521- I suggest to cut this sentence and replace with “We have described our context and results in rich detail which will allow readers to assess the transferability of findings to their setting.” It is important to discuss appropriate strengths and applications of qualitative research.
6. **Throughout**
   a. There is inconsistency in terms re: communicable disease versus infectious disease. I suggest to eliminate one of the two.
