# Supplementary material for: The feasibility of respondent-driven sampling with people who use drugs in rural Western Cape, South Africa: A qualitative study
Source: PLOS Glob Public Health. 2025 Jun 26;5(6):e0004065. doi: 10.1371/journal.pgph.0004065 (PMC12200662; doi:10.1371/journal.pgph.0004065)
Supplement: Attachment — Submitted filename: response to reviewers May 2025.docx [file pgph.0004065.s009.docx]

**Responses to Reviewer Comments**

The authors would like to thank the reviewer for their final detailed read of the submitted manuscript as well as careful consideration of the revisions made. We are very pleased that the reviewer now feels with some minor additional changes that the manuscript meets all of the criteria for publication in PLOS Global Health. Detailed responses to issues raised have been provided in the table below:

**Reviewer 1:**

| **Abstract** | **Response to Reviewer** |
| --- | --- |
| 1. Line 52. Phrasing of the sentence is awkward. I suggest cutting “to conduct formative research” as it is not necessary to the meaning of the sentence and the word formative is mentioned in the conclusion. | We appreciate the careful review of the abstract, and are in agreement with the reviewer, and have deleted this part of the sentence. |
| **Introduction** |  |
| 1. Line 103-108. The use of incentives remains unclear. I suggest the authors rephrase these lines to more clearly explain. Do all who recruit additional participants receive incentives? At what point of the process? If this varies for different studies, that could be described. 2. Line 114- 129 – It remains is difficult to follow this paragraph as the requirements for RDS and the connection between this study and the main study are mixed together. For clarity, I suggest to move some of the sentences from lower in the paragraph to the start. 3. Line 130 – the population for the study is not clearly described in the research question. I suggest to rephrase research question to “we aimed to explore the perceptions of PWSD in a rural region of the Western Cape on the feasibility and appropriateness of participation in a research studying using RDS. 4. Line 139 – cut the word possibly- typo? | We have added some additional information to this section of the introduction but would like to highlight that a major difference between RDS and traditional recruitment is that while incentives are provided to all research participants successful peer recruitment means additional incentives. In addition, the median number included in the introduction is based on a systematic review as a recommendation, but I have added that it can vary depending on study needs. Additional information has been added to line 108-114.  This has been moved to the start of the paragraph as suggested.  We thank the reviewer for this suggestion on rephrasing the research question,and have revised this appropriately from line 139-144.  We have changed this to “probably”-as we cannot assume that this is most definitely the case but it is likely. |
| **Methods** |  |
| 1. Line 164- Sentence starting “participants were …” is long and confusing. I suggest breaking up by stating “Participant were recruited to the study in three ways. First… 2. Line 183- Transcription and translation practices are not clearly described. I suggest “FGDs were transcribed verbatim in Afrikaans and isiXhosa by staff who spoke those languages. Afrikaans and isiXhosa transcripts were then translated into English    i. Unless there was back translation to Afrikaans and isiXhosa, I suggest cutting the word back from this sentence.    ii. What training was provided to the original transcribers? 3. Line 191 – I suggest rephrasing to “perspectives on study methods (e.g., peer recruitment, biomedical fingerprints)”. 4. Line 195- Please describe the amount of incentive 5. Analysis – Line 199 – 211 – methods for condensing codes into higher order themes are not described. Please add practices used to complete analysis. | The methods of recruitment have been numbered for easier reading between line 177 and 178.  The authors thank the reviewer for this careful read of the transcription and translation process and have modified this for clarity, as well as added detail on the original transcribers, in line 196-198.  This has been revised and now reads RDS and planned study activities in line 204-205, since peer recruitment in RDS is almost always followed by also biobehavioral data collection, and fingerprinting is essentially part of the recruitment process.  The incentive amount for focus group participation has been added in the local currency, namely South African rands.  This had now been added in the data analysis section as suggested (see line 220-221). |
| **Results** |  |
| 1. Line 227- the word “characteristics” is repeated. Please cut one for clarity. 2. Line 325 – 337 – Although the stigmatized nature of drug use is an important consideration for this study, the authors do not describe the impact of this stigma on research participation. Please add one or two sentences to explain this link. | We would like to thank the reviewer for reading this so thoroughly and have cut the second one.  A sentence has now been added to line 362-363 to explain why stigma is important for this group of participants, and how it could affect their study participation. |
| **Discussion** |  |
| 1. Sentence at line 482- as per point above. This is not clearly supported by the data. Further description of the results on this topic would address this. 2. Line 520-521- I suggest to cut this sentence and replace with “We have described our context and results in rich detail which will allow readers to assess the transferability of findings to their setting.” It is important to discuss appropriate strengths and applications of qualitative research. | This has been addressed by adding the sentence above (in results section).  Thank you, we have reworked this sentence in the discussion to rather frame this in a positive way, and feel that due to the reviewer’s suggestion the transition to the next paragraph on study strengths now reads clearly. |
| **Conclusion** |  |
| 1. There is inconsistency in terms re: communicable disease versus infectious disease. I suggest to eliminate one of the two. | Although both terms are correct-as TB is a communicable, infectious disease-we have decided to use the term infectious disease for consistency and to be consistent with the prevalent literature. |
