# Supplementary material for: The feasibility of respondent-driven sampling with people who use drugs in rural Western Cape, South Africa: A qualitative study
Source: PLOS Glob Public Health. 2025 Jun 26;5(6):e0004065. doi: 10.1371/journal.pgph.0004065 (PMC12200662; doi:10.1371/journal.pgph.0004065)
Supplement: Attachment — Submitted filename: PLOS RDS re-review May 19_nb.docx [file pgph.0004065.s010.docx]

May 19, 2025

Dear Drs Kyobutungi and Pai

Thank you for this opportunity to re-review the manuscript titled "The feasibility of respondent-driven sampling (RDS) with people who smoke drugs (PWSD) in rural Cape Town, South Africa: a qualitative study”. In this work, the authors describe a preliminary study conducted to gather the perspectives of PWSD in the Western Cape of South Africa on participating in research which incorporates RDS. With the latest revisions, the authors have addressed all concerns previously raised. However, I suggest they conduct one final review amongst the team to address the remaining typographical errors, awkward phrases, and run-on sentences. I offer the authors my warm congratulations for their work on this topic which will lead to improved health for an important and underserved population.
